# Measurement properties of PROMIS short forms for pain and function in total hip arthroplasty patients

**DOI:** 10.1186/s41687-021-00313-1

**Published:** 2021-05-30

**Authors:** Anika Stephan, Vincent A. Stadelmann, Michael Leunig, Franco M. Impellizzeri

**Affiliations:** 1 Department of Teaching, Research and Development – Lower Extremities, Schulthess Clinic, Lengghalde 2, 8008 Zürich, Switzerland; 2 Hip Surgery, Schulthess Clinic, Lengghalde 2, 8008 Zürich, Switzerland; 3 Faculty of Health, University of Technology Sydney, PO Box 123, Broadway, Ultimo, NSW 2007 Australia

## Abstract

**Introduction:**

While the Patient-Reported Outcomes Measurement Information System (PROMIS) is mainly designed for computer adaptive testing, its static short forms (SF) are used when a paper-pencil format is preferred or item banks are not yet translated into the target language. This study examined the measurement properties of the German PROMIS-SF for pain intensity (PAIN), pain interference (PI) and physical function (PF) in total hip arthroplasty (THA) patients.

**Methods:**

SF were collected before and 12 months post-surgery. Higher scores indicate more PAIN, higher PI and better PF. Oxford Hip Score (OHS) was the main reference measure. Six months post-surgery, a subsample completed the SF twice within 14 days to test reliability.

**Results:**

Of 172 eligible patients, 147 consented to participate and received questionnaires; 132 (74 males) returned baseline questionnaires (mean age 65.8 ± 10.2 years) and 116, 12-month questionnaires. Forty-five patients provided test-retest data.

Correlations of all SF with OHS were large (│r│ ≥ 0.7; confidence intervals did not include 0.50). Cronbach’s alpha values were: PAIN, 0.86; PI, 0.93; PF, 0.91. Intraclass correlation coefficients were: PAIN, 0.77; PI, 0.81; PF, 0.69. Standard errors of measurement were: PAIN, 3.8; PI, 2.8; PF, 3.6. Smallest detectable change thresholds were: PAIN, 8.8; PI, 6.6; PF, 8.4. Follow-up data showed a ceiling effect (best score) for PAIN (66%), PI (76%), and PF (66%). SF change scores showed large correlations with OHS change scores (│r│ > 0.6).

**Conclusion:**

Our results provide some evidence of construct validity, and acceptable reliability and responsiveness of PROMIS-SF for pain and function in THA patients. These SF can thus be considered acceptable for use, although patients’ improvement in physical function might be underestimated due to the large follow-up PF score ceiling effects.

## Plain English summary

Measurement qualities of PROMIS instruments are mainly assessed for computer adaptive testing but not for non-adaptive short questionnaires. As these questionnaires are in use, their measurement properties must also be evaluated. Results from computer adaptive testing cannot simply be transferred.

We studied the measurement qualities of the German PROMIS short questionnaires for pain intensity, pain interference and physical function in patients undergoing hip replacement. We wanted to see how these questionnaires perform when compared to the Oxford Hip Score, a standard questionnaire commonly used to test hip-related disability in these patients.

The three questionnaires can be considered acceptable for use in hip replacement patients, but some limitations do exist. Patient improvement in physical function might be underestimated because many patients reach the highest possible score and further improvements cannot be measured. Also, any small but important improvement in physical function cannot be distinguished from measurement error in individual patients.

## Introduction

The Patient Reported Outcomes Measurement Information System (PROMIS®) aims to provide a common health metric for many medical conditions [1]. It is primarily designed for computer adaptive testing (CAT). However, PROMIS static short forms (SF) are also available and in use. PROMIS measurement properties have been investigated in total hip arthroplasty (THA) patients [2–5] but are mostly limited to CAT and focused on single aspects of validity [2], interpretability [4, 5] or responsiveness [3]. Conversely, the measurement properties of PROMIS-SF for pain and function in THA patients remain largely undetermined.

German language CAT item banks for pain and function were under development by the German PROMIS group at the time of this study. In future, these PROMIS CAT instruments will be offered by this group for third party use via REDCap (personal communication). Furthermore, not all patients actually prefer electronic over paper forms (according to an internal survey where we found half of our patients reporting their preference for paper questionnaires), and this can influence response rate and adherence. The SF can be easily implemented in clinical registries (especially the shortest versions), while connecting CAT platforms to active registries might initially require additional resources. We decided to use the shortest available SF, which were most feasible for our purposes and minimized respondent and administrative burden (i.e. potential barriers to the collection of patient-reported measures in a clinical setting and registries). Therefore, the aim of the study was to examine the psychometric properties of German PROMIS-SF for pain intensity (PAIN), pain interference (PI) and physical function (PF) in THA patients. Valid SF would allow the use of PROMIS metrics when CAT cannot be implemented or when SF are deemed more feasible.

## Materials and methods

### Study design and questionnaire administration

**Fig. 1:**
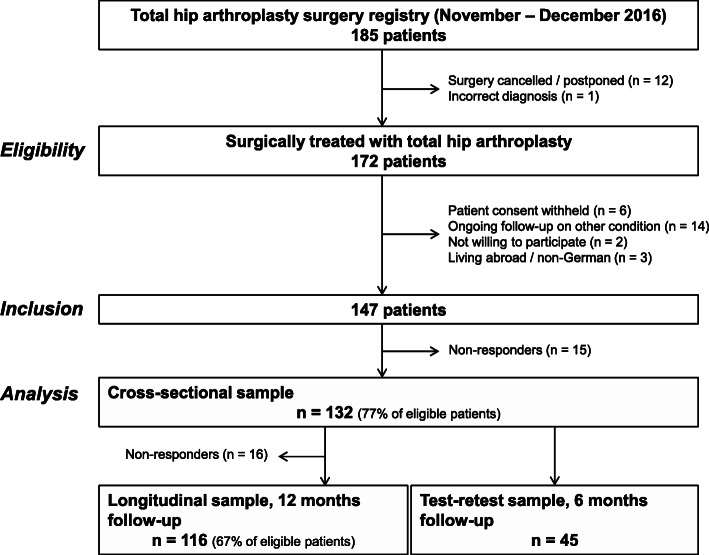
Flowchart showing patient eligibility and sample sizes for assessing German PROMIS short form measurement properties

This prospective study included consecutive patients of our THA registry from November and December 2016 (Fig. 1). Enrolled patients had to provide consent to use their data for research purposes. Exclusion criteria were living abroad, insufficient knowledge of the German language, cognitive impairment or ongoing follow-up of former surgeries. Ethics approval was obtained. Patient-reported outcomes were collected from paper questionnaires administered 1 to 4 weeks before (baseline) and again, from paper questionnaires or, if chosen by the patient, via online survey 12 months after surgery. A subsample of consecutive patients completed questionnaires 6 months after surgery with a retest occurring within 14 days (median: 6 days) for reliability testing until a sample size of 30 was reached. The patients’ condition was considered as stable in this period.

### Outcome questionnaires

We investigated PROMIS-SF for PAIN (3 items), PI and PF (each with 4 items) provided by the PROMIS Germany research group. Answers are given on 5-point verbal rating scales. For PAIN, we used the form 3a (v2.0) that assesses pain over a 7-day recall period and current pain [6]. Form 4a (v1.0) defined PI based on the consequences of pain on relevant aspects of one’s life over a 7-day recall period [7, 8]. For PF, we used form 4a (v2.0) [9, 10] assessing the current ability to perform various physical activities. Overall scores for PAIN, PI and PF were presented as T-scores; higher scores indicate more PAIN, higher PI and better PF. A score of 50 (10) represents the US general population mean (standard deviation). Scoring was done by using the “HealthMeasures Scoring Service”, powered by Assessment Center^SM^. Missing items were not replaced.

We used the reference Oxford Hip Score (OHS), a condition-specific instrument that assesses constructs encompassing the selected PROMIS domains and 2 single-item questions rating surgical success.

Specifically, we used the cross-culturally adapted and validated German OHS [11, 12]. This 12-item, joint-specific self-administered questionnaire is valid, reliable and responsive for assessing pain and disability in THA patients. Items are answered on 5-point Likert scales extending from 0 to 4 points, where 4 indicates the best outcome. Total scores, calculated by adding all items, range from 0 (worst) to 48 points (best). OHS was shown to have a two-factor structure (pain, function) as well [13].

At 12 months, patients rated their global treatment outcome (GTO): “*How much did the operation help your hip problem?*” on a 5-point Likert scale ranging from “helped a lot” to “made things worse” [14]. They also defined their state of symptom-specific well-being (SSWB): “*If you had to spend the rest of your life with the symptoms you have right now, how would you feel about it?*” on a 5-point Likert scale ranging from “very satisfied” to “very dissatisfied” [15].

### Evaluation of measurement properties

Construct validity was assessed using scale-specific hypothesis testing and considered good if at least 75% of the hypotheses were confirmed. We tested correlations with OHS total score and OHS pain and function subscales at baseline and 12 months, and SSWB at 12 months. All correlations were expected to be large (confidence intervals ≥0.5), and specific correlations were expected to be negative for PAIN and PI with OHS and for PF with SSWB and positive for PAIN and PI with SSWB and PF with OHS.

Internal consistency was calculated using Cronbach’s alpha with values between 0.70 and 0.95 indicating appropriate internal consistency [16]. Test-retest reliability was assessed with the intraclass correlation coefficient (ICC) from a single measurement, absolute agreement, 2-way mixed-effects model; an ICC (confidence interval) ≥ 0.7 was considered acceptable [16]. Agreement was assessed using the standard error of measurement (SEM_agr_ = √(variance due to systematic differences between measurements + residual variance)). The effect size based on SEM_agr_ was calculated from the mean change score. The smallest detectable change (SDC) for individuals that can be considered above the measurement error with a 90% confidence level was calculated as SDC90 = 1.65 * √2 * SEM_agr_ [17].

Responsiveness defines the ability of a questionnaire to detect clinically important changes over time. Longitudinal validity can be considered a measure of responsiveness and is examined by inspecting the correlation between change scores of the instrument under validation and the reference instrument. We expected negative correlations between change scores of PAIN, PI and OHS, and positive correlations between change scores of PF and OHS, each in the order of |r| (confidence intervals) ≥ 0.5. The smallest effect size of interest was defined as a Cohen’s *d* ≥ 1.5 for the decrease in PI and increase in PF based on other studies [3, 18]. Responsiveness was considered sufficient if at least 75% of the hypotheses were confirmed.

Floor and ceiling effects were considered acceptable if percentages were below 15%. To determine the individual-level minimal important change (MIC), we used linear regression with the OHS change scores and reported MIC for OHS in THA patients [19].

Analyses were performed using Stata Statistical Software Release 15 (StataCorp LP, TX, USA).

## Results

Table 1 presents the baseline demographics with pain and function status. Age range was 32 to 93 years with a median of 66.8 years. Most surgeries were primary THA (92%) and 8% of patients underwent THA revisions.

**Table 1:** Baseline patient characteristics and score changes

Characteristics^a^	Cross-sectional (***N*** = 132)	Longitudinal (***N*** = 116)	Test-retest (***N*** = 45)
Age (years)	65.4 (10.9)	65.9 (10.2)	68.5 (10.9)
Sex (female) (n, %)	58 (43.9)	51 (44.0)	16 (35.6)
Height (cm)	171.1 (9.1)	171.5 (8.9)	171.5 (9.0)
Weight (kg)	78.4 (14.8)	77.9 (14.9)	79.4 (13.3)
Body mass index (kg/m^2^)	26.7 (4.0)	26.4 (3.9)	26.9 (3.6)
PROMIS PAIN (T-score)	65.7 (8.7)	65.0 (8.8)	64.2 (9.6)
PROMIS PI (T-score)	64.4 (7.0)^b^	64.2 (7.2)	63.9 (8.3)
PROMIS PF (T-score)	36.9 (5.5)^b^	37.3 (5.5)	37.2 (6.0)
OHS	22.1 (8.7)^c^	22.5 (8.8)^d^	22.5 (9.9)
PROMIS PAIN (T-score change, 95% CI)		−20.0 (−21.6 to −18.4)	
PROMIS PI (T-score change, 95% CI)		−19.6 (−21.1 to −18.0)	
PROMIS PF (T-score change, 95% CI)		15.8 (14.5 to 17.0)	
OHS (score change, 95% CI)		22.6 (21.0 to 24.3)	

*PROMIS* Patient Reported Measurement Outcome Instrumentation System, *PAIN* Pain intensity, *PI* Pain interference, *PF* Physical function, *T-score* Overall PROMIS score calculated per domain, *OHS* Oxford Hip Score, *CI* Confidence interval

^a^Expressed as mean with standard deviation unless otherwise stated

^b^For one case, two of four items were missing, but score calculation was still possible with automated response pattern scoring

^c^For five cases, one to two items were missing and replaced by the mean of all other items to calculate a score

^d^One item was missing for one case, which was replaced by the mean of all other items to calculate a score

### Construct validity

Scale-specific hypothesis testing for validity resulted in 100% confirmed hypotheses for PAIN, 89% for PI and 78% for PF (Table 2).

**Table 2:** Correlations between PROMIS scales and OHS and SSWB

	Correlation with OHS^**a,b**^	Correlation with SSWB^**a,c**^
PROMIS PAIN		
Baseline	−0.83 (− 0.88 to -0.77)	–
	−0.84 (−0.88 to −0.78)^e^	
12 months	− 0.84 (−0.89 to −0.78)	0.69 (0.58 to 0.77)
	−0.86 (−0.90 to −0.80)^e^	
Change	−0.81 (−0.87 to -0.74)^d^	–
PROMIS PI		
Baseline	−0.84 (−0.89 to −0.78)	–
	−0.78 (−0.81 to −0.71)^e^	
12 months	−0.84 (−0.88 to −0.77)	0.66 (0.55 to 0.76)^g^
	−0.81 (−0.86 to −0.74)^e^	
Change	−0.76 (−0.82 to −0.67)^d^	–
PROMIS PF		
Baseline	0.82 (0.75 to 0.87)	–
	0.80 (0.73 to 0.86)^f^	
12 months	0.79 (0.70 to 0.85)	−0.53 (−0.65 to −0.38)^h^
	0.80 (0.72 to 0.86)^f^	
Change	0.72 (0.63 to 0.80)^d^	–
OHS		
12 months	–	−0.65 (−0.74 to −0.53)
Change	–	–

*PROMIS* Patient Reported Measurement Outcome Instrumentation System, *OHS* Oxford Hip Score, *SSWB* Symptom-specific well-being, *PAIN* Pain intensity, *PI* Pain interference, *PF* Physical function

^a^Correlation coefficients are shown for the total sample. Analysis was done for male and female subsamples as well. If not indicated (^g^), correlation coefficients for male and female subsamples are above the required threshold (confidence intervals do not include 0.5)

^b^Pearson’s correlation coefficient (r)

^c^Spearman’s rank correlation coefficient (r_s_)

^d^Correlation OHS change score

^e^Coefficients for OHS pain subscale

^f^Coefficients for OHS function subscale

^g^Large correlation was not confirmed for the male subsample (absolute values of the confidence interval limits were required to be ≥0.5)

^h^Large correlation was not confirmed for the total sample and male subsample (absolute values of the confidence interval limits were required to be ≥0.5)

### Reliability

Cronbach’s alpha ranged between 0.7 and 0.95. ICC confidence intervals were ≥ 0.7 for PAIN and PI, but not for PF (Table 3). PAIN showed the highest SEM_agr_ and SDC90, whereas PI had the lowest. The effect size based on SEM_agr_ was smallest for PF, and smaller than OHS for all three SF.

**Table 3:** Reliability, agreement and smallest detectable change

	Cronbach’s α^a^	ICC^a^	SEM_**agr**_	SDC90	$\frac{\mathbf{mean}\ \mathbf{change}\ \mathbf{score}}{{\mathbf{SEM}}_{\mathbf{agr}}}$
PROMIS PAIN	0.86 (0.82 to 0.90)	0.77 (0.60 to 0.87)	3.75	8.75	6.4
PROMIS PI	0.93 (0.90 to 0.94)	0.81 (0.67 to 0.89)	2.83	6.61	6.9
PROMIS PF	0.91 (0.88 to 0.93)	0.69 (0.50 to 0.81)	3.60	8.40	4.4
OHS	–	–	2.08	4.85^b^	10.9

*ICC* Intraclass correlation coefficient, *SEM*_*agr*_ Agreement assessed using standard error of measurement, *SDC90* Smallest detectable change for individuals that can be considered above the measurement error with a 90% confidence level, *PROMIS* Patient Reported Measurement Outcome Instrumentation System, *PAIN* Pain intensity, *PI* Pain interference, *PF* Physical function, *OHS* Oxford Hip Score

^a^95% confidence interval in parentheses

^b^Beard et al. (2015) [19]

### Responsiveness

**Fig. 2:**
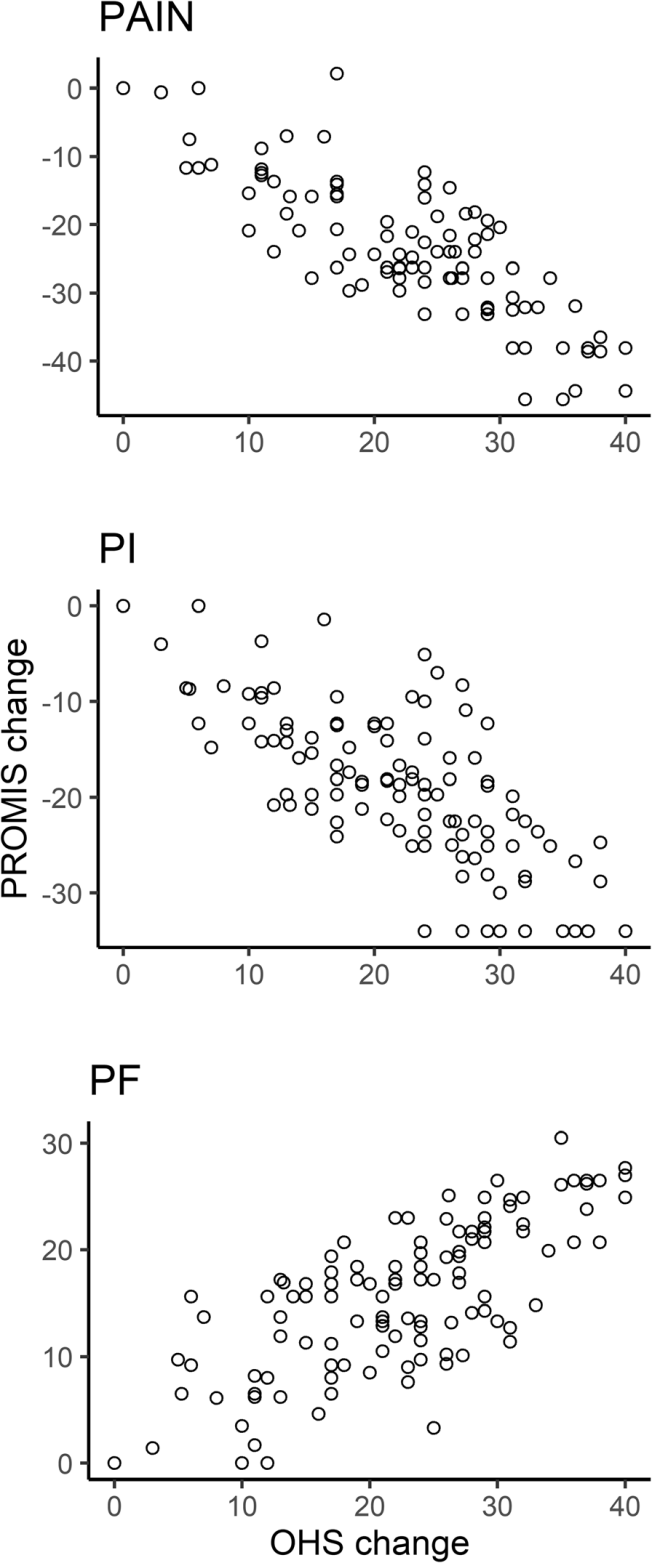
Responsiveness plots

Hypothesis testing for responsiveness resulted in confirmation of all hypotheses about correlations between SF and OHS change scores; SF change score scatter plots are shown in Fig. 2. We observed a cluster of cases with a PI change score of − 34, which represents patients that changed from the worst to best PI score (14 of 116 cases). Cohen’s *d* (95% confidence interval) values were: PAIN, − 2.9 (− 3.3 to − 2.5); PI, − 3.0 (− 3.4 to − 2.6); PF, 2.7 (2.4 to 3.1).

We found ceiling effects (best score) for PAIN (66%), PI (76%), and PF (66%) after surgery. MICs were: PAIN, − 10; PI, − 8.8; PF, 7.2 (T-score change).

## Discussion

Our results suggest that the construct validity of PROMIS-SF is acceptable in THA patients. The SF have good internal consistency, test-retest reliability and responsiveness. For PAIN and PI, MICs were larger than the corresponding SDC90 values. Some measurement property limitations were nevertheless detected.

For PF, MIC was smaller than SDC90 meaning that clinically relevant change could not be distinguished from measurement error on the individual level. Compared to OHS, all SF show 40% to 60% smaller effect sizes based on SEM_agr_, which means that the joint-specific OHS allows more detailed grading of patient recovery than the PROMIS-SF scales.

The high proportion of patients with best possible scores of PI and PAIN after surgery may be not critical. These scales represent unipolar constructs where the complete absence of pain or pain interference makes it difficult (yet likely less relevant) to differentiate them any further. Nevertheless, researchers should be careful in interpreting PF after surgery because of ceiling effects. This problem may be resolved by using PF CAT without substantially increasing respondent burden [20, 21]. Although confirmation of this aspect is warranted, we think it is unlikely that longer SF (i.e. 6b, 8b, 20a or 12a for people who can walk) will impact the ceiling effect because their maximum T-score is only slightly higher (59 to 66) than that of the 4-item SF (57) [9, 21]. There was also 12% of patients who went from the worst possible to best possible PI score from baseline to follow-up, which can be critical if a more detailed grading of recovery is desired.

### Limitations

THA is typically associated with very high patient satisfaction. Consequently, we did not have patients in the “poor outcome” category upon dichotomisation of the GTO, and MIC could not be calculated with an anchor-based standard method using the receiver operating characteristics curve. For this reason, we adopted an alternative indirect approach using linear regression from the OHS MIC calculated in a much larger study with 82,415 THA patients [19].

Only 77% of eligible patients responded at baseline and 67% at follow-up. From our internal registry quality-control procedures, we know that “lack of time” is the most common reason for not responding. From follow-up non-responders, less than 3% refused to cooperate because they were dissatisfied with their treatment, which suggests that there was no major selection bias.

Unidimensionality of the SF scale structure was not assessed, due to existing reports and guidelines of the development of PROMIS item banks [1, 22, 23]. The unidimensionality of the PF and PI item banks has been reported previously [8, 24].

## Conclusion

Our results provide some evidence of construct validity, and acceptable reliability and responsiveness of PROMIS-SF for pain and function in THA patients. The SF can thus be considered as acceptable as another common static instrument (i.e. OHS) for use in these patients, although improvement in PF might be underestimated due to the large follow-up PF score ceiling effects.

## Data Availability

The datasets used and/or analysed during the current study are available from the corresponding author on reasonable request.

## Abbreviations

CAT: Computer adaptive testing
GTO: Global treatment outcome
ICC: Intraclass correlation coefficient
MIC: Minimal important change
OHS: Oxford Hip Score
PAIN: Pain intensity
PF: Physical function
PI: Pain interference
PROMIS: Patient Reported Outcomes Measurement Information System
REDCap: Research electronic data capture
SDC: Smallest detectable change
SEMagr: Agreement assessed using the standard error of measurement
SF: Short forms
SSWB: Symptom-specific well-being
THA: Total hip arthroplasty
